# Resident to exhausted CD4^+^ T cell ratio is associated with the prognosis of gastric cancer

**DOI:** 10.1016/j.gendis.2023.101076

**Published:** 2023-09-07

**Authors:** Jinxiang Lv, Libo Wang, Wenlong Jia, Hui Xu, Siyuan Weng, Yuyuan Zhang, Zhe Xing, Shuang Chen, Shutong Liu, Yuhao Ba, Changqing Guo, Zaoqu Liu, Xinwei Han

**Affiliations:** a Department of Interventional Radiology, The First Affiliated Hospital of Zhengzhou University, Zhengzhou, Henan 450052, China; b Interventional Institute of Zhengzhou University, Zhengzhou, Henan 450052, China; c Interventional Treatment and Clinical Research Center of Henan Province, Zhengzhou, Henan 450052, China; d Department of Gastroenterology, The First Affiliated Hospital of Zhengzhou University, Zhengzhou, Henan 450052, China; e Department of Hepatobiliary and Pancreatic Surgery, The First Affiliated Hospital of Zhengzhou University, Zhengzhou, Henan 450052, China; f Hepatic Surgery Center, Tongji Hospital, Tongji Medical College, Huazhong University of Science and Technology, Wuhan, Hubei 430030, China; g Clinical Medical Research Center of Hepatic Surgery at Hubei Province, Wuhan, Hubei 430030, China; h Department of Neurosurgery, The Fifth Affiliated Hospital of Zhengzhou University, Zhengzhou, Henan 450052, China; i Center for Reproductive Medicine, The First Affiliated Hospital of Zhengzhou University, Zhengzhou, Henan 450052, China; j School of Basic Medical Sciences, College of Medicine, Zhengzhou University, Zhengzhou, Henan 450052, China; k State Key Laboratory of Proteomics, Beijing Proteome Research Center, National Center for Protein Sciences (Beijing), Beijing Institute of Lifeomics, Beijing 102206, China; l State Key Laboratory of Medical Molecular Biology, Institute of Basic Medical Sciences, Chinese Academy of Medical Sciences, Department of Pathophysiology, Peking Union Medical College, Beijing 100730, China

Gastric cancer (GC) ranks fifth for cancer incidence and fourth for mortality globally.^1^ Clinical outcomes have varied among patients receiving similar treatments at the same stage, suggesting the current prognostic tools remain somewhat flawed.^2^^,^^3^ Single-cell analysis of GC data allowed us to dissect transcriptional programs underlying lymphocyte residency and exhaustion. Combined with tumor purity, we developed a novel classification system and data analysis following the workflow shown in Figure S1.

A total of 116,704 cells were divided into 33 clusters (Fig. 1A) and predominantly consisted of 9 clusters (Fig. S2A). Specific markers were analyzed to confirm the accuracy of cell populations (Fig. S2B). Further, according to CD4^+^ T cell markers including IL7R, STAT4, AAK1, CD2, RPL7, and RPS6, as well as CD8^+^ T cell-specific signature genes GZMA, GZMB, IFNG, GZMH, NKG7, and PRF1, we annotated T cell clusters 0, 5, and 7 as CD4^+^ T cells, while clusters 1 and 21 were identified as CD8^+^ T cells (Fig. 1B; Fig. S2C).

**Figure 1:**
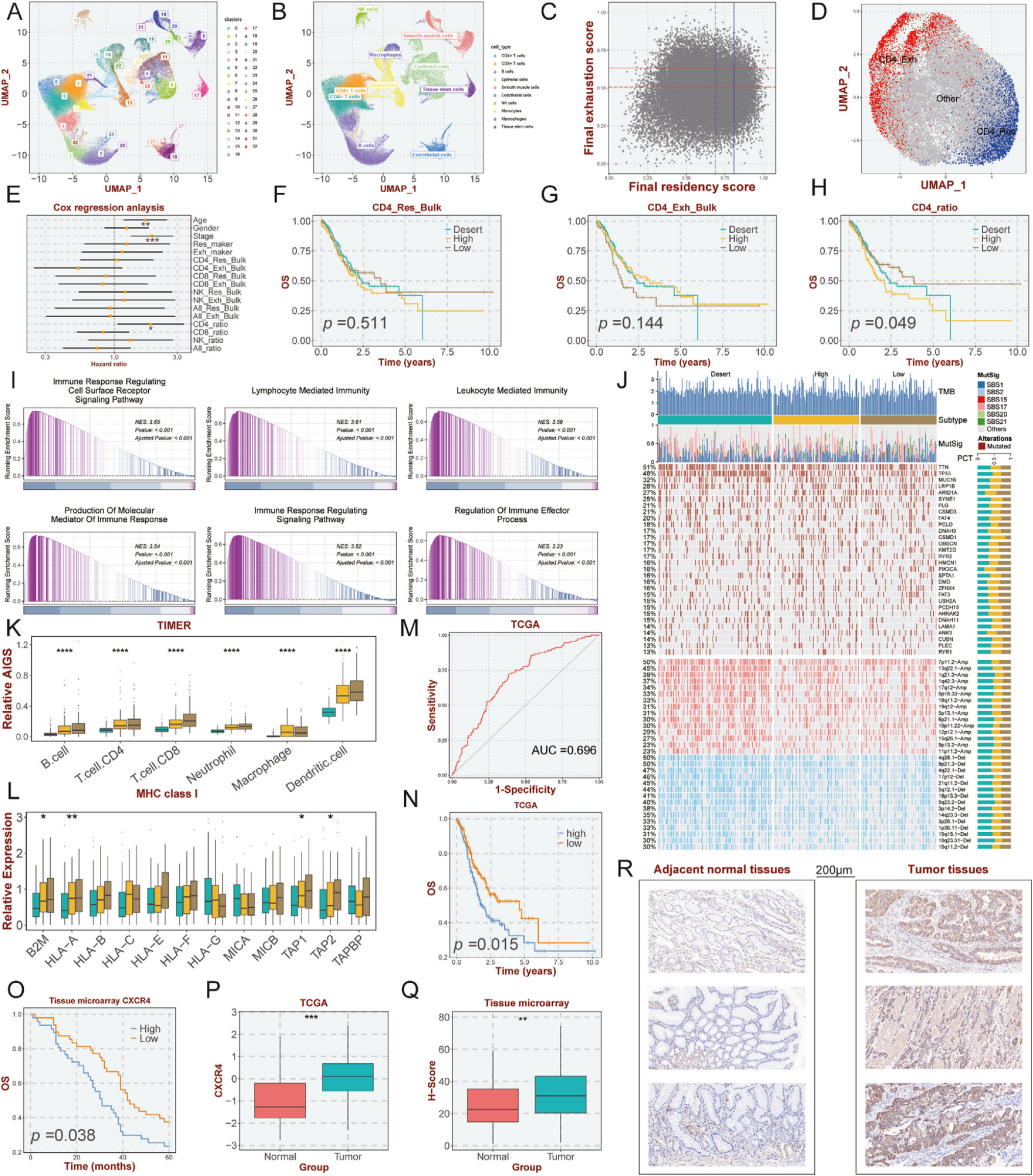
Development of resident to exhausted CD4^+^ T cell ratio and immunohistochemical verification of tissue chip. **(A, B)** Uniform Manifold Approximation and Projection (UMAP) plot of all the single cells, with each color coded for major cell clusters and subpopulations. **(C, D)** Final residency and exhaustion scores and cell clusters in each of the CD4^+^ T cells. The solid lines identify exhausted cells, while the dashed lines identify resident cells. **(E**–**H)** Multivariate COX regression analysis and survival analysis. **(I**–**L)** Functional enrichment analysis, mutational landscapes, immune infiltration abundance, and distribution of MHC I molecules among three clusters. **(M**–**R)** Detection of potential biomarkers for GC patients and validation in TCGA and tissue microarray cohorts.

A significant concentration of canonical resident and exhausted markers was found in the CD4^+^ T, CD8^+^ T, and NK cell areas (Fig. S3A, B). To determine initial resident and exhausted signatures, we computed correlations between selected canonical resident and exhausted markers and previously retrieved genes (Table S1). We then quantified the relative concordance of individual cells with initial signatures by *singscore* package. According to the scoring threshold, we divided cells into resident cells (high residency and low exhaustion scores), exhausted cells (high exhaustion and low residency scores), and others. Next, the genes that differed between resident and exhausted cells were filtered by percentile thresholds. We estimated the score in each cell again based on the resultant genes (Fig. 1C; Fig. S3C). Moreover, we compiled the final grouping of resident and exhausted cells in CD4^+^ T, CD8^+^ T, and NK cells. Resident and exhausted cells were robustly separated (Fig. 1D; Fig. S3D).

As suggested by Foroutan et al,^4^ we filtered genes that were over-expressed in tumor cells by a “bulk threshold” to ensure accuracy in the definition of infiltrating immune cells, and reassigned genes that overlapped among three cell types based on their correlation between signature scores and gene expression in each cell type. Thus, bulk RNA-sequencing from tumor samples can identify resident and exhausted programs using the final signatures [CD4_Res_Bulk (*n* = 27), CD4_Exh_Bulk (*n* = 24), CD8_Res_Bulk (*n* = 26), CD8_Exh_Bulk (*n* = 45), NK_Res_Bulk (*n* = 141), and NK_Exh_Bulk (*n* = 99)]. Furthermore, we merged all resident and exhausted signatures respectively for general analysis [All_Res_Bulk (*n* = 194), All_Exh_Bulk (*n* = 168); Table S2].

A total of 348 GC samples were left for prognostic analysis in TCGA-STAD after 31 paired normal samples were removed. To further examine the prognostic value of the above signatures along with tumor purity, we conducted a multivariate Cox regression analysis (Fig. 1E). It was determined that age (>65 years), stage (III-IV *vs*. I-II), and a higher ratio of CD4^+^ T cells were independent risk factors for overall survival in GC patients. Further, after dividing GC patients into three groups based on tumor purity and residency-to-exhaustion ratio of the three cells, Kaplan-Meier survival analysis demonstrated that only the CD4_ratio was significantly related to overall survival (Fig. 1F–H; Fig. S4A–D). Patients with a low CD4_ratio possessed a favorable prognosis, whereas those with a higher CD4_ratio had poorer outcomes.

To decode the representative biological features among the three groups, we performed GSEA based on gene sets from Hallmark, GO, and KEGG. The desert group exhibited conspicuous enrichment in ribosome biogenesis, chromosome segregation, rRNA metabolic process, DNA replication, cell cycle checkpoint signaling, and methyltransferase complex (Fig. S5A). Meanwhile, calcium-mediated signaling, sprouting angiogenesis, positive regulation of MAPK cascade, ERK1/2 cascade, epithelial to mesenchymal transition, and cell signaling by WNT were enriched in the high CD4_ratio group (Fig. S5B). The low CD4_ratio group was significantly associated with immune-related pathways, for instance, lymphoid-mediated immunity, leukocyte-mediated immunity, immune response regulating signaling pathway, production of molecular mediator of the immune response, regulation of immune effector process, and cell activation involved in immune response (Fig. 1I).

The three groups were further analyzed in terms of genomic variation. Information about the top 30 frequently mutated genes and top 15 commonly amplification and deletion regions was presented, along with an overview of mutational signatures and tumor mutation burden (Fig. 1J). Mutation percentages of the majority of mutant genes tended to be highest in the desert group. Further investigation of chromosomal instability revealed that the selected regions of chromosome amplification and loss accounted for the highest proportions in the desert group with statistically significant. The tumor mutation burden quantification analyses revealed the desert group had the highest tumor mutation burden (*P* = 0.0058) (Fig. S6A). Immunophenoscore was most prominent in the desert group, which may be related to mutations (Fig. S6B). Additionally, the fraction genome altered, fraction genome gain, fraction genome loss, focal gain, focal loss, arm gain, and arm loss were significantly conspicuous in the desert group, indicating a highly genomic and chromosomal instability (Fig. S6C–I).

We sought to examine the differences among the three groups in respect of immune cells. Figure 1K illustrated the greatest number of immune cells observed in the low CD4_ratio group. Consistently, MHC I, MHC II, co-inhibitory, and co-stimulatory molecules were also highest in the low CD4_ratio group (Fig. 1L; Fig. S7A–C). Taking into account the findings of Thorsson and colleagues,^5^ we analyzed the proportions of the six classical immunotypes in the three groups. C2 (IFN-γ dominant, with favorable prognosis) was more prominent in the low CD4_ratio group, whereas C1 (wound healing, with a high proliferation rate) dominated in the desert group (Fig. S7D). In addition, the low CD4_ratio group exhibited the highest scores on several pivotal immune characteristics including T cell receptor richness, T cell receptor Shannon, B cell receptor richness, B cell receptor Shannon, leukocyte fraction, and lymphocyte infiltration signature score (Fig. S7E–J). In terms of proliferation, the desert group ranked highest, while the high CD4_ratio group earned the highest scores as to TGF-β responses (Fig. S7K, L).

Given the darkest survival outcome of patients in the high CD4_ratio group, we sought to develop an ideal biomarker to serve clinical practice. Eventually, we identified a chemokine receptor, CXCR4, which could accurately identify patients with a high CD4_ratio in the TCGA-STAD cohort (AUC = 0.696; Fig. 1M). The result from the Kaplan-Meier curve for the cohort indicated that increased CXCR4 expression was related to observably worse overall survival (Fig. 1N). Additionally, we further validated the above results at the protein level using the immunohistochemistry staining experiment with a tissue microarray including tumor and adjacent normal tissues. Further, 47 patients were categorized as the high CXCR4 expression group and 48 patients as the low CXCR4 expression group in accordance with median value, and patients with low CXCR4 expression owned remarkably prolonged overall survival (*P* = 0.038; Fig. 1O). The expression levels of CXCR4 were noticeably increased compared with the normal tissues in the TCGA-STAD cohort (Fig. 1P). Consistently, our experiment further confirmed the results (Fig. 1Q, R). Our study highlighted the prognostic significance and potential biomarker possibilities of CXCR4 in GC.

In conclusion, our model including the ratio of CD4^+^ T resident and exhausted signatures and tumor purity provided a practical and effective way to assess GC patient survival outcomes and might facilitate clinical management.

## Author contributions

ZQL and XWH provided direction and guidance throughout the study. JXL, LBW, and WLJ analyzed data and wrote the manuscript. LBW collected the in-house cohort, while WLJ and JXL collected data from the public database. ZQL, LBW, and CQG reviewed and made significant revisions to the manuscript. HX, SYW, YYZ, ZX, SC, STL, and YHB collected and prepared the related papers. All authors read and approved the final manuscript.

## Conflict of interests

The authors declare that they have no competing interests.

## Funding

This study was supported by the Major Science and Technology projects of Henan Province, China (No. 221100310100).
