# Supplementary material for: Resident to exhausted CD4+ T cell ratio is associated with the prognosis of gastric cancer
Source: Genes Dis. 2023 Sep 7;11(4):101076. doi: 10.1016/j.gendis.2023.101076 (PMC10955202; doi:10.1016/j.gendis.2023.101076)
Supplement: Multimedia component 1 [file mmc1.docx]

**Supplementary figures**

**
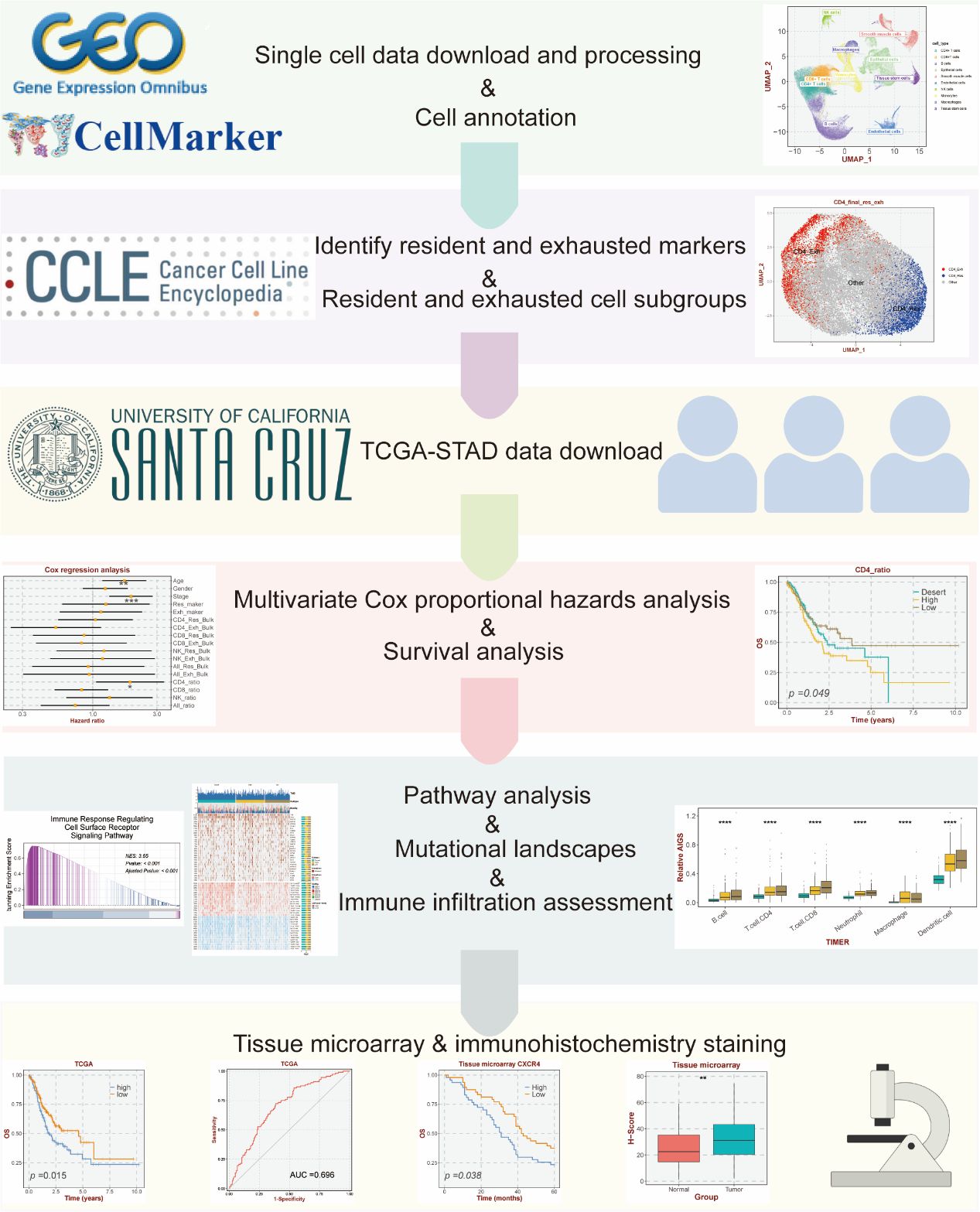
**

**Figure S1. Workflow of our analysis.**


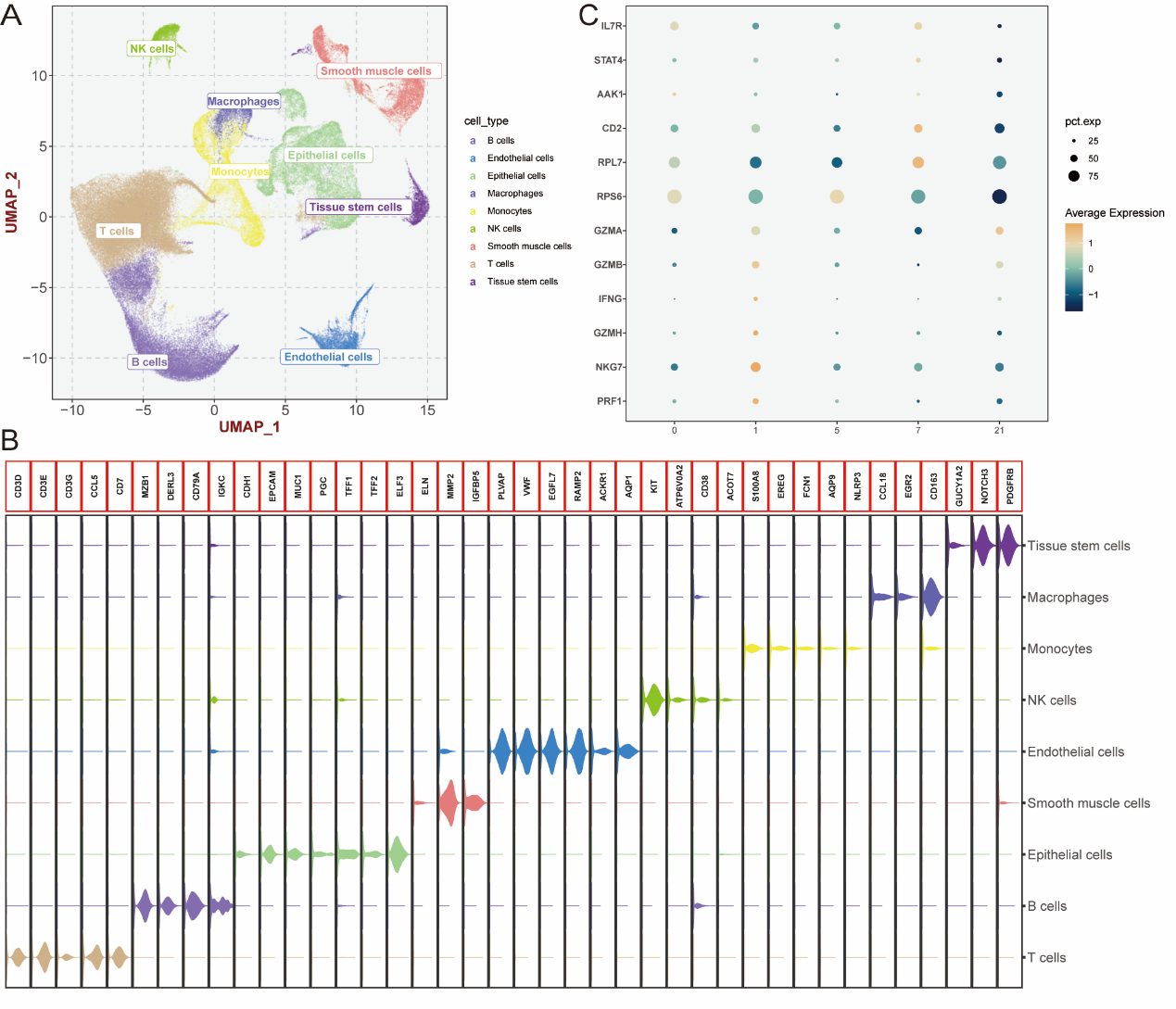


**Figure S2. The clustering analysis of single cell data for gastric cancer.** (A) Uniform Manifold Approximation and Projection (UMAP) plot of all the single cells, with each color coded for 9 cell types. (B) Marker genes of 9 cell type. (C) Marker genes of CD4+ T cells and CD8+ T cells.


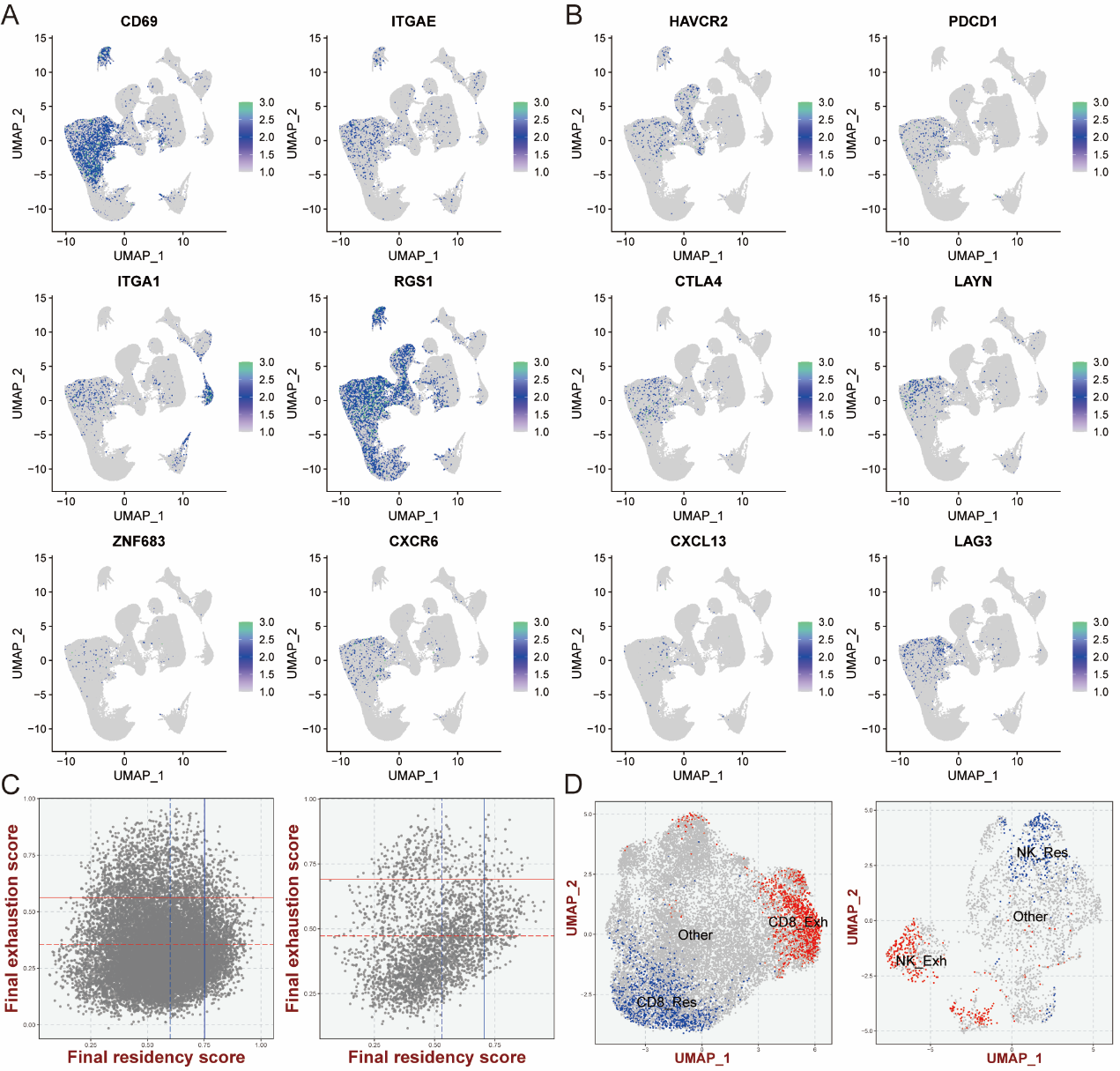


**Figure S3.** **UMAP plots of canonical residency and exhaustion genes and clusters of resident and exhausted cells.** (A, B) UMAP plots showing the expression level of canonical residency genes and canonical exhaustion genes. (C) Final residency and exhaustion scores in each of the CD8+ T cell, and NK cell. The solid lines identify exhausted cells, while dashed lines identify resident cells. (D) Using marker genes to cluster resident cells, exhausted cells, and others in each of cell populations.


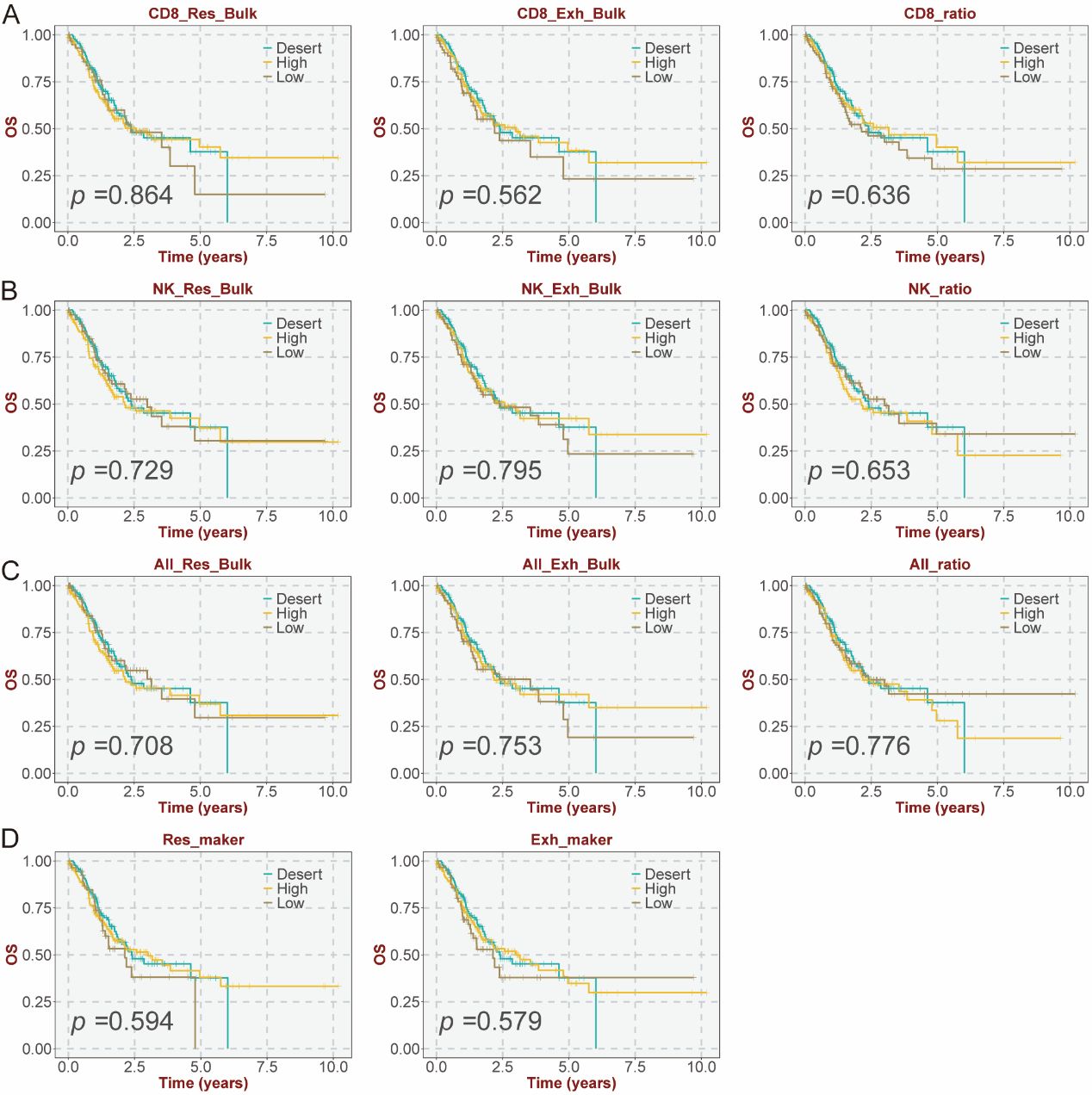


**Figure S4.** **Kaplan–Meier analysis.** (A-D) Kaplan-Meier curves for OS among different groups. *P*-values were obtained using the log-rank test.


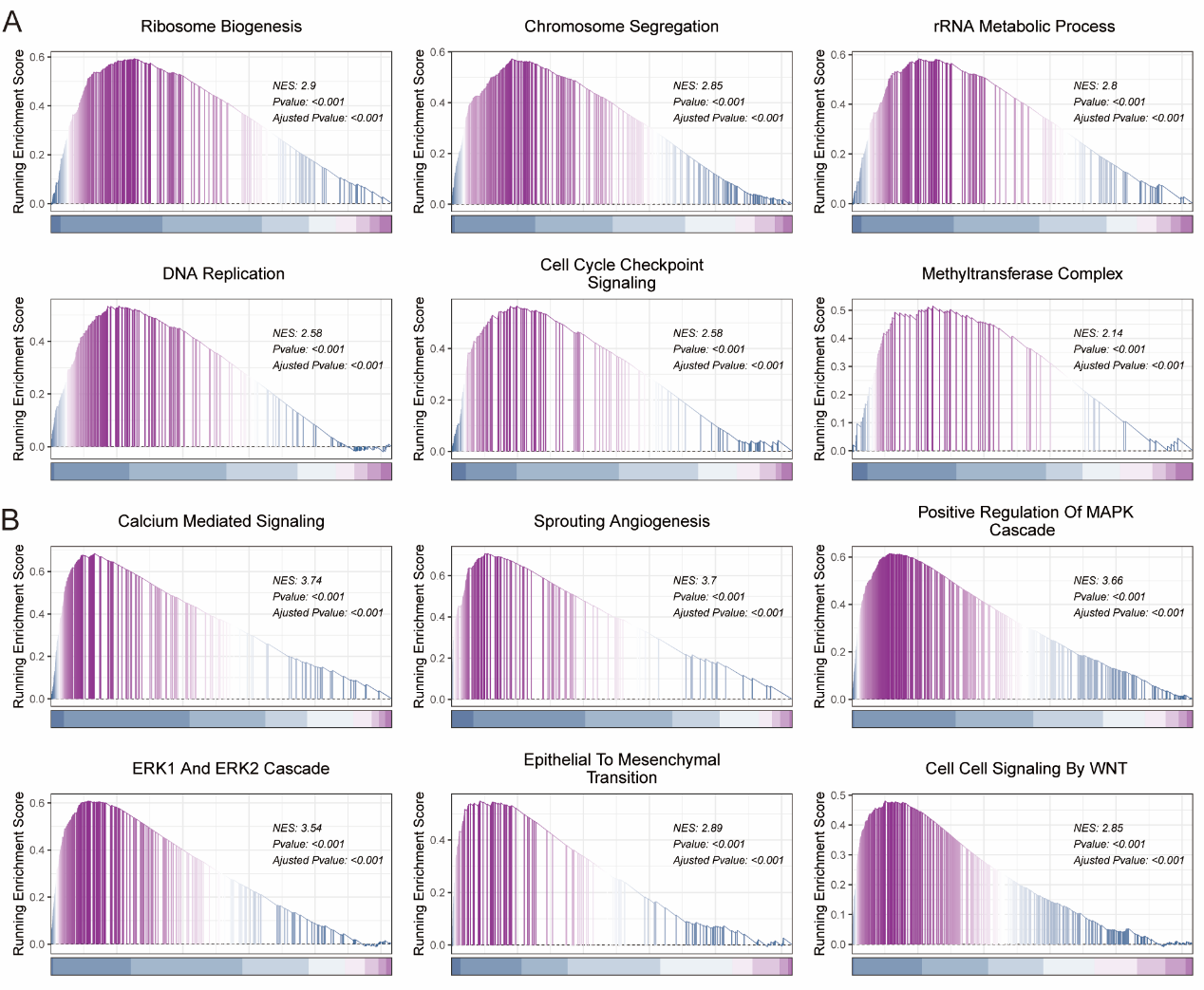


**Figure S5.** **Gene set enrichment analysis.** (A, B) Enrichment terms depicted by gene set enrichment analysis based on Gene Ontology for the desert and the high CD4 ratio groups, respectively.


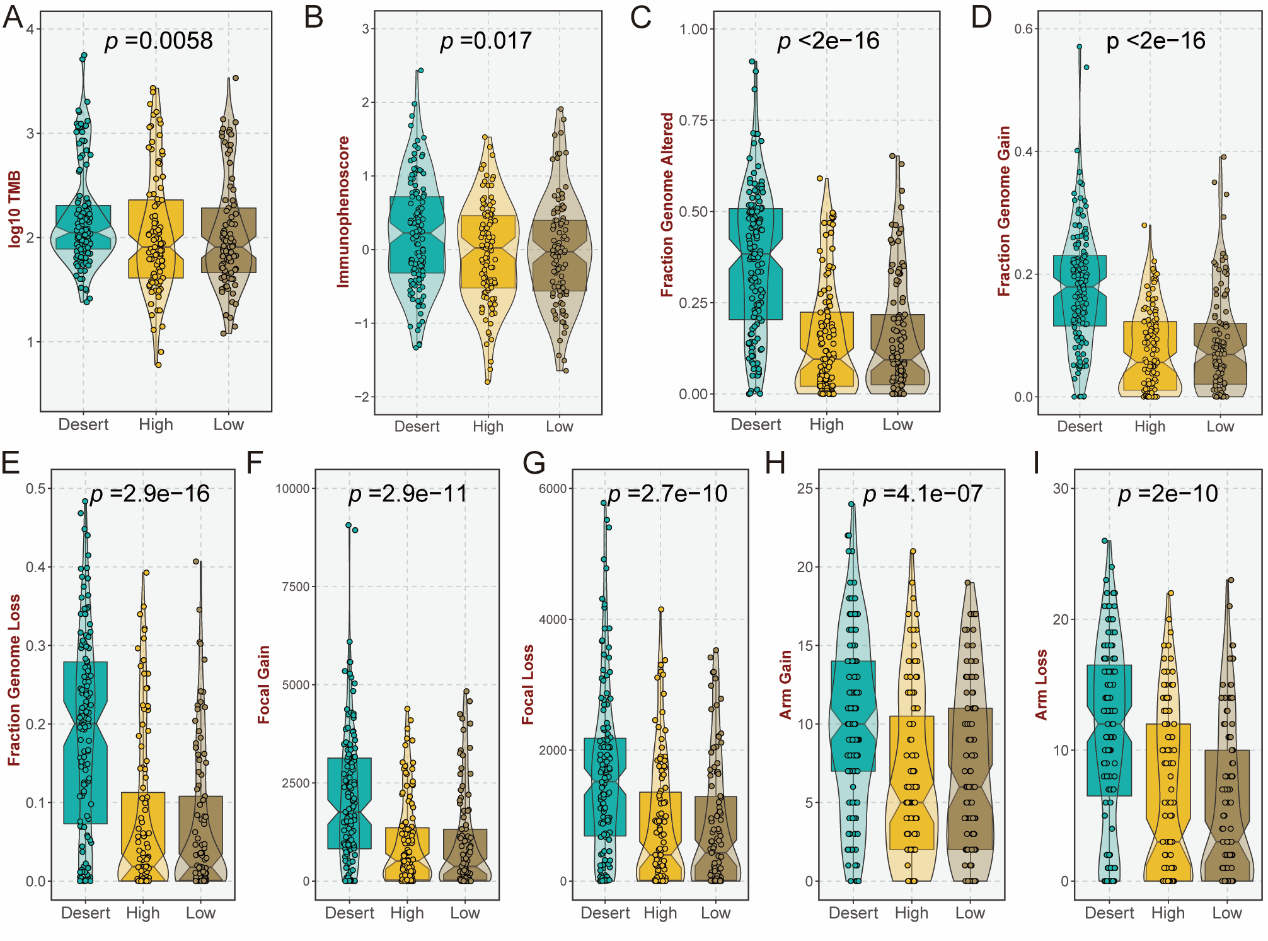


**Figure S6. Characteristics of genomic variations among three groups.** (A-I) We compared tumor mutation burden (TMB) (A), immunophenoscore (B), the fraction genome altered (C), fraction genome gain (D), fraction genome loss (E), focal gain (F), focal loss (G) arm gain (H), and arm loss (I) among three groups.


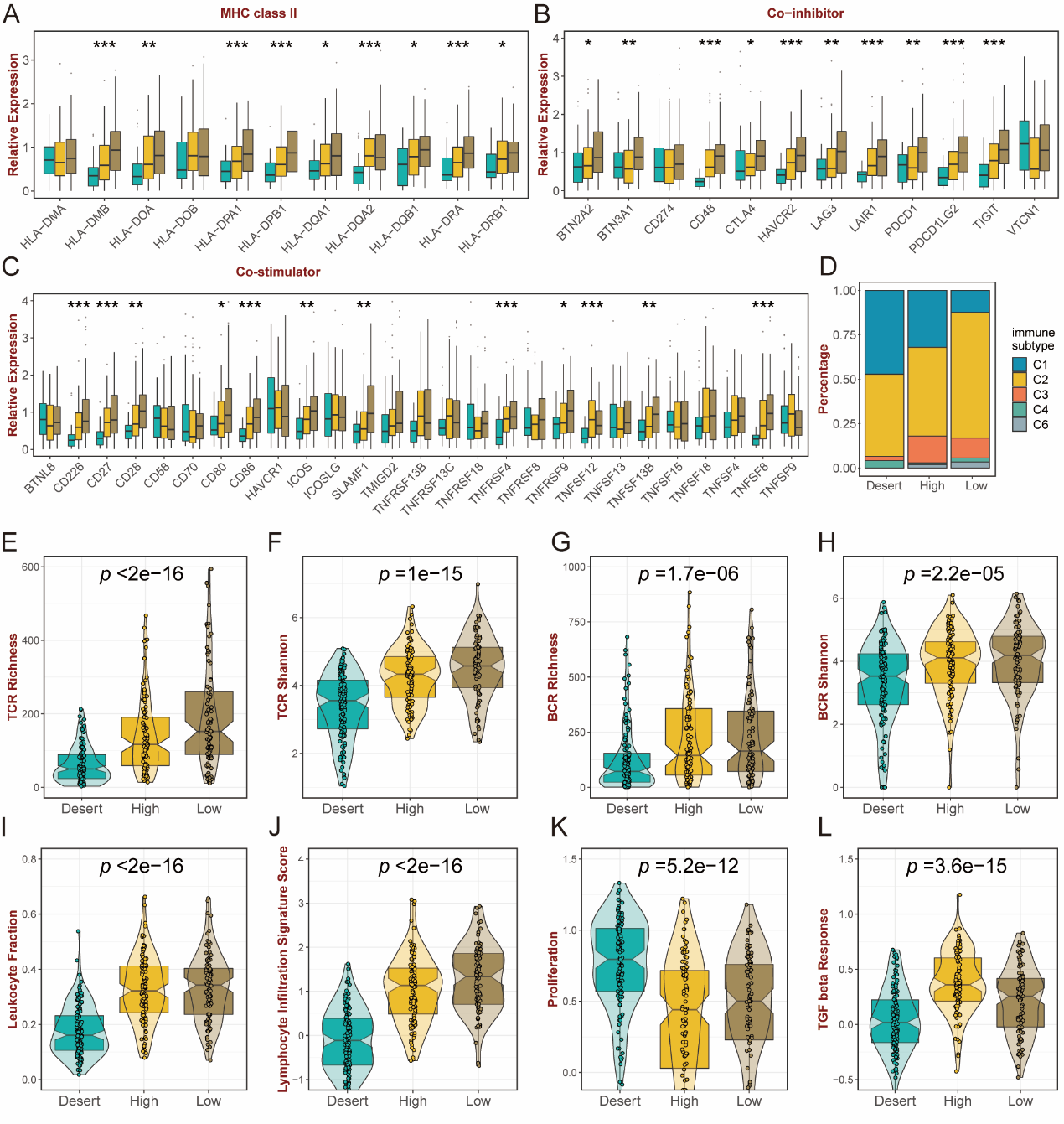


**Figure S7. Immune landscape among three groups.** (A-C) Distribution of MHC II (A), co-inhibitory (B), and co-stimulatory (C) molecules among three clusters. (D) Proportions of 6 clusters among three groups. (E-L) Other immune characteristics among three clusters.
