# Supplementary material for: Resident to exhausted CD4+ T cell ratio is associated with the prognosis of gastric cancer
Source: Genes Dis. 2023 Sep 7;11(4):101076. doi: 10.1016/j.gendis.2023.101076 (PMC10955202; doi:10.1016/j.gendis.2023.101076)
Supplement: Multimedia component 2 [file mmc2.docx]

**Methods**

**Single-cell RNA sequencing (scRNA-seq) data analysis**

Raw count files for scRNA-seq were retrieved from GEO (<https://www.ncbi.nlm.nih.gov/geo/>) under accession number GSE183904[1]. The dataset covered 40 samples, with 10 normal primary gastric tissue samples, 1 normal peritoneum sample, 26 tumor primary gastric tissue samples, and 3 tumor peritoneum samples. Aiming to investigate the lymphocyte levels within the tumor tissue, we selected the 26 gastric cancer (GC) tissue primary specimens for analysis. Following quality control according to the standard procedure of *Seurat* package (V4.3.0), we annotated the different clusters resulting from reduced-dimension clustering by *SingleR* package (V1.10.0) based on the reference dataset Human Primary Cell Atlas data which was downloaded via the *celldex* software package (V1.6.0). We used the FindAllMarkers module to detect genes expressed in over 25% of the cells within each cluster. Furthermore, T cells were classified as CD4+ and CD8+ T cells using markers acquired from the CellMarker database (<http://biocc.hrbmu.edu.cn/CellMarker/>). According to the distribution of 6 residency canonical markers genes (CD69, ITGAE, ITGA1, RGS1, ZNF683, CXCR6) and 6 exhaustion canonical markers genes (HAVCR2, PDCD1, CTLA4, LAYN, CXCL13, LAG3) in scatter plots of the Uniform Manifold Approximation and Projection (UMAP), CD4+ T, CD8+ T, and NK cells were selected for subsequent studies.

**Establishing the** **initial residency and exhaustion signatures**

A comprehensive list of resident and exhausted genes was extracted from previous literature **(Table S1)**. Expression information extraction of the extensive gene lists was performed independently in three cell types. Resident canonical markers we used were: CD69, ITGAE, RGS1, CXCR6 for CD4+ T cells, the same genes plus ZNF683 for CD8+ T cells, and CD69, ITGAE, ITGA1, RGS1, ZNF683, CXCR6 for NK cells. Exhausted canonical markers were consisted of HAVCR2, PDCD1, LAYN, CXCL13 for CD4+ T cells, PDCD1, CTLA4, LAYN for CD8+ T cells, HAVCR2, PDCD1, CTLA4, LAYN, CXCL13, LAG3 for NK cells. We calculated Spearman correlations (*p*) between canonical markers and genes from the list for each cell separately. As a result of various distributions of gene expression in each cell, these thresholds diversified significantly. For CD4+ T cells, genes with *p* >0.20 with the residency canonical markers were considered resident markers, and those with *p* >0.15 with the exhausted canonical genes were counted as exhausted markers. For CD8+ T cells, genes with *p* >0.25 with the residency canonical markers and *p* >0.20 with the exhausted canonical genes were regarded as resident markers and exhausted markers, respectively. For NK cells, the correlation coefficient thresholds were set to 0.30 and 0.25, then genes above the threshold were characterized as resident markers and exhausted markers, individually. The overlap between the resident markers and exhausted markers was removed. In each of the cell populations, canonical correlation analysis was carried out and a coefficient <-0.1 in the first component was enforced to reduce the correlation between the resident markers and exhausted markers. As a result, the remaining resident markers and exhausted markers were preserved for further analysis as initial sets.

**Establishing the final residency and exhaustion signatures**

Against initial residency and exhaustion signatures, CD4+ T, CD8+ T, and NK cells were scored by the *singscore* package (V1.16.0). Based on initial residency and exhaustion scores, the cells were layered into resident cells, exhausted cells, and others. Cells with residency scores above the 90th percentile and exhaustion scores below the 60th percentile were resident cells, and a similar threshold was set for exhausted cells. Differentially expressed genes (DEGs) were acquired between resident cells and exhausted cells through the Wilcoxon rank sum test and the Model-based Analysis of Single-cell Transcriptomics method. Genes satisfying adjusted *p* <0.05 and log2 fold change (logFC) >0.5 remained. In accordance with Foroutan *et al.*[2], further refined genes were identified by comparing the transcript abundance percentile of resident and exhausted cells. We retained resident genes whose 65th percentile expression in resident cells was superior to the 90th percentile abundance in exhausted cells, and refinement of exhausted genes was also consummated using analogous comparisons. In particular, NK signatures were defined using the 65th and 80th percentile threshold. Final signatures of residency and exhaustion were generated from the resultant gene sets.

**Single-cell data reprocessing**

We reprocessed CD4+ T, CD8+ T, and NK cells separately. Principal component analysis using final residency and exhaustion signatures was carried out to reduce the dimensionality. Inspecting the elbow plot, we chose the top 10 principal components (PCs) for CD4+ T cell clustering and the top 20 PCs for CD8+ T cell and NK cell clustering. By clustering each cell type using the FindClusters function, we obtained ultima scatter plots of the resident and exhausted populations for three cell types and presented them by UMAP.

**Refinement of the signatures based on cell line and bulk data**

Gene expression data for multiple personal cancer cell lines were obtained from the cancer cell line encyclopedia (CCLE) (<https://portals.broadinstitute.org/ccle/>). Through the *edgeR* package (V3.38.4), a DGEList object was generated, whose genes with count-per-million (CPM) >1 in at least 5 cell lines were filtered. Following the Trimmed Mean of M-values (TMM) normalization, the Reads Per Kilobase Million (RPKM) values were calculated. Subsequently, we only visualized the markers genes expressed in the GC cell lines. Resident and exhausted signatures passed the “bulk tumor threshold” if their median logRPKM expression ≤ the 75th percentile for GC cell lines.

RNA-seq data for TCGA-STAD (n =379) were acquired from the UCSC Xena (<http://xena.ucsc.edu/>). For each sample, single sample Gene Set Enrichment Analysis (ssGSEA) was used to calculate enrichment scores, quantifying marker genes passing the “bulk tumor threshold” and canonical resident and exhausted marker genes. Based on the correlation between scores of three cell types and expression information, we reassigned the overlapping genes accordingly. Ultimately, the relocated genes were suitable for bulk data analysis **(Table S2)**.

**Grouping and Survival analysis of TCGA bulk data**

Using ssGSEA algorithm, we calculated the proportion of resident and exhausted scores among the CD4+ T, CD8+ T, and NK cell populations, respectively. Afterward, based on the estimate scores obtained by ESTIMATE algorithm, we further calculated tumor purity and stratified patients into the desert group (high tumor purity group) as well as high (high residency to exhaustion score) and low ratio groups (low residency to exhaustion score). The prognostic performance of the resulting measures was estimated by Cox regression and Kaplan-Meier survival curves.

**Functional enrichment analysis**

Using gene sets from Hallmark, Gene Ontology (GO) and Kyoto Encyclopedia of Genes and Genomes (KEGG) downloaded from MSigDB (<https://www.gsea-msigdb.org/gsea/msigdb>), we conducted GSEA for DEGs among the desert group, the high CD4_ratio group, and the low CD4_ratio group. GSEA scores were computed and visualized by the *clusterProfiler* package (V4.4.4) and the *GseaVis* package (V0.0.4), respectively.

**Immune infiltration assessment**

We performed a comprehensive immune infiltration analysis of the desert, the high CD4_ratio, and the low CD4_ratio patients by integrating TIMER, CIBERSORT, CIBERSORT-ABS, xCell, QUANTISEQ, MCPCOUNTER, and EPIC methods. We also compared co-stimulatory, co-inhibitory, MHC I, and MHC II molecules extracted from previous reports among the three subtypes[3]. Additionally, comparisons were also conducted with several vital indicators including immune subtypes, proliferation, TGFβ responses, lymphocyte infiltration signature score, leukocyte fraction, T-cell receptor (TCR) richness, TCR Shannon, B-cell receptor (BCR) richness, and BCR Shannon[4].

**Mutational landscapes**

We downloaded somatic mutation and [masked copy number segment](https://xenabrowser.net/datapages/?dataset=TCGA-STAD.masked_cnv.tsv&host=https%3A%2F%2Fgdc.xenahubs.net&removeHub=https%3A%2F%2Fxena.treehouse.gi.ucsc.edu%3A443) of TCGA-STAD from UCSC Xena. Gene and region information of copy number alternation (CNA) for TCGA-STAD was obtained from the Firebrowse database (<http://firebrowse.org/>). The *deconstructSigs* package (V1.8.0) and *NMF* (V0.24.0) package were used to calculate mutational signature distribution. The top 30 hypervariable genes calculated by the *maftools* package (V2.12.0) were visualized as genes for CNA. For the CNA regions, our criteria were areas with significant differences among the three groups, and CNA amplification or deletion frequencies in the top 15. Furthermore, we compared tumor mutation burden (TMB), immunophenoscore, the fraction genome altered, fraction genome gain, fraction genome loss, and copy number gain and loss burden in chromosomal and focal regions among the three groups.

**Tissue microarray and immunohistochemistry staining**

We purchased STAD Human tissue microarrays (HStmA180Su30) from Shanghai Outdo Biotechnology, Shanghai, China. The clinical characteristics of 97 STAD and 83 adjacent normal tissue specimens were gathered from the company websites. Anti-CXCR4 (750 μg/ml: Cat No. 11073-2-AP, Proteintech, Wuhan, China) antibody was used for immunohistochemistry. A histochemistry score (H-score) that combined staining intensity and percentage of intensity was used to semiquantitate the expression of the target molecule in tissue microarray, with a larger H-score representing a higher expression.

**Statistical analysis**

R (V4.2.1) was used to generate all statistical analyses and graphs. Correlations between continuous variables were calculated using Spearman correlation analysis. A chi-squared or Fisher exact test and a T-test or Wilcoxon rank-sum test were carried out to compare categorical and continuous variables, respectively. Multiple subtypes were compared using the ANOVA or Kruskal-Wallis test. *P* <0.05 was perceived as statistically significant.

1. Kumar V, Ramnarayanan K, Sundar R, Padmanabhan N, Srivastava S, Koiwa M, et al. Single-Cell Atlas of Lineage States, Tumor Microenvironment, and Subtype-Specific Expression Programs in Gastric Cancer. Cancer Discov. 2022; 12: 670-91.

2. Foroutan M, Molania R, Pfefferle A, Behrenbruch C, Scheer S, Kallies A, et al. The Ratio of Exhausted to Resident Infiltrating Lymphocytes Is Prognostic for Colorectal Cancer Patient Outcome. Cancer Immunol Res. 2021; 9: 1125-40.

3. Liu Z, Zhang Y, Shi C, Zhou X, Xu K, Jiao D, et al. A novel immune classification reveals distinct immune escape mechanism and genomic alterations: implications for immunotherapy in hepatocellular carcinoma. J Transl Med. 2021; 19: 5.

4. Thorsson V, Gibbs DL, Brown SD, Wolf D, Bortone DS, Ou Yang TH, et al. The Immune Landscape of Cancer. Immunity. 2018; 48: 812-30 e14.
